# Ill, Itinerant, and Insured: The Top 20 Users of Emergency Departments in Baltimore City

**DOI:** 10.1100/2012/726568

**Published:** 2012-04-19

**Authors:** Barbara Y. DiPietro, Dana Kindermann, Stephen M. Schenkel

**Affiliations:** ^1^Department of Public Policy, Health Care for the Homeless, 421 Fallsway, Baltimore, MD 21202, USA; ^2^Department of Emergency Medicine, Georgetown University Hospital/Washington Hospital Center, 110 Irving Street, NW, Washington, DC 20010, USA; ^3^Department of Emergency Medicine, University of Maryland School of Medicine, 110 South Paca Street, Suite 200, Baltimore, MD 21201, USA

## Abstract

The purpose of this study was to document the clinical and demographic characteristics of the 20 most frequent users of emergency departments (EDs) in one urban area. We reviewed administrative records from three EDs and two agencies providing services to homeless people in Baltimore City. The top 20 users accounted for 2,079 visits at the three EDs. Their mean age was 48, and median age was 51. Nineteen patients visited at least 2 EDs, 18 were homeless, and 13 had some form of public insurance. The vast majority of visits (86%) were triaged as moderate or high acuity. The five most frequent diagnoses were limb pain (*n* = 9), lack of housing (*n* = 6), alteration of consciousness (*n* = 6), infection with human immunodeficiency virus (HIV) (*n* = 5), and nausea/vomiting (*n* = 5). Hypertension, HIV infection, diabetes, substance abuse, and alcohol abuse were the most common chronic illnesses. The most frequent ED users were relatively young, accounted for a high number of visits, used multiple EDs, and often received high triage scores. Homelessness was the most common characteristic of this patient group, suggesting a relationship between this social factor and frequent ED use.

## 1. Introduction

Frequent emergency department (ED) users present unique challenges to clinicians and hospital administrators. Several studies have examined the impact of this patient group on ED utilization [1–4]. This population places significant economic, time, and space burdens on EDs. Patients who come to an ED more than three times per year represent about 7% of total ED users [1]. They tend to be in poor physical and mental health, and they often come to an ED for treatment of acute medical problems [2–4]. Although the definition of “frequent user” varies greatly, distinct utilization characteristics have been noted, including a high rate of use of other parts of the health care system and visiting multiple EDs [5].

 Most studies have examined the characteristics of ED visits from a macrolevel, but this study narrows the focus to the patient-specific level. We examined the details of visits made by the 20 most frequent users of three EDs in Baltimore City in 2005 to describe the clinical and demographic characteristics of this group. The study is distinct in its attempt to look at the cooccurrence of homelessness and very high ED utilization patterns.

Approximately 1% of the US population experiences homelessness each year, and homeless patients represent about 0.4% of all ED users [6, 7]. One qualitative study from 1998 found that frequent ED users had a high prevalence of homelessness. In that study, approximately 70% of heavy users who were interviewed were homeless and many users admitted to seeking both medical and nonmedical relief during their ED visits [8].

 This study seeks to more fully describe the visit details of a complex patient group, focusing on a social variable that likely contributes to high ED utilization. In particular, it strives to provide a perspective of the experience of homelessness among Baltimore City's most frequent ED users.

## 2. Methods

This was a retrospective study based on a review of administrative records from three EDs located within 2 miles of each other and of the databases maintained by two agencies providing services to homeless people in the same urban area of Baltimore City. The most frequent users of the EDs were identified. Patients were considered homeless if they had come to at least one of the service agencies during the study year (2005). The study focuses on the 20 most frequent users identified in the combined dataset.

Each of the 20 patients was assigned a unique identifier by combining parts of the individual's social security number, date of birth, and gender and race codes. In previous research, this methodology for creating a unique identifier has been demonstrated to be compliant with requirements of institutional review boards (IRBs) and 99.8% unique to an individual [9]. These identifiers allowed patient-specific analysis of ED utilization while protecting patient confidentiality. The same methodology was used to assign identifiers to all clients of the city's location of Health Care for the Homeless during 2005 and to individuals represented in the city's centralized data system that captures utilization of publicly funded shelter beds and daytime drop-in service centers. A patient was considered homeless for the purposes of this study if his/her identifier was found in one of the homeless services administrative databases.

 The top 20 most frequent users were identified based on total ED visits at all three institutions. Information regarding total number of ED visits, number of EDs visited, and health insurance was extracted from the ED records. Triage scores as well as the most frequently occurring ICD-9 codes listed for each patient visit were recorded. The number of visits receiving “high,” “moderate,” or “low” triage scores were summed, with a “high” score correlating to triage level 1 or 2, “moderate” to level 3, and “low” to level 4 or 5. Finally, the diagnostic codes for each patient were compiled and tallied across all visits for each patient.

 The study was approved by the institutional review board at each participating hospital and at the university with which the lead author was affiliated at the time of the study.

## 3. Results

During calendar year 2005, the number of patient visits at the three EDs totaled 159,340. The top 20 users made 2,079 visits during this time, accounting for 1.3% of the total.  Table 1 details the demographic, clinical, and housing status characteristics of these 20 patients. The average age in this group was 48.3 years, and the median age was 51 (range, 23–68). The top seven users each visited an ED at least 100 times during the year. Most of the 20 users visited all three EDs (*n* = 13), 6 individuals used two EDs, and 1 went to one facility. The majority of patients had either Medicaid (*n* = 8) or Medicare (*n* = 4), 7 were uninsured, and one was noted as both having Medicaid and being uninsured. The most common characteristic among these top 20 users was homelessness: 18 (90%) had contact with homeless service agencies during the year.

 For each patient, the top five most prevalent ICD-9 codes (calculated to the prime decimal point for each three-digit category) were tallied (Table 1). The ICD-9 codes indicate that some patients presented frequently for the same reasons, and others had a wide range of codes, indicating a lesser degree of consistency across visits. For example, one patient's five top diagnostic codes represented 39% of that patient's codes; for another, they represented 93% of all codes. Most patients had a relatively narrow range of diagnoses, with the top five accounting for the majority of reasons for their visits. Overall, the group's conditions span a range of acute and chronic disease (Table 2). Of the diagnoses that applied to at least 4 of the 20 patients, the most common was limb pain (9 patients).

## 4. Discussion

We examined the clinical and demographic characteristics of a subset of ED users who account for a disproportionately large number of total ED visits. The most frequent users are relatively young, in poor health, and experience homelessness in high numbers. The 20 patients identified in this study accounted for more than 2,000 ED visits in 2005, constituting just over 1% of the total visits for these three EDs during the study period. While our local area has housed a small number of long-term homeless individuals in a supported housing program since 2005, the general economic downturn since that time and related increase in the area's homeless population would likely make our findings more striking today.

The sheer number of visits is likely to have imposed significant time, space, and resource burdens on the EDs. A quarter of visits by the study population received high triage scores, and another 60% received moderate scores. Although triage scores are an imperfect proxy for severity of illness, this scoring pattern suggests that frequent users are relatively ill.

 Most users cycled among the three EDs: 95% visited at least two of them, and 65% used all three. The sharing of electronic medical records among the hospitals might allow better coordination of the care being delivered, reduce the possibility of conflicts in medications or discharge instructions, and limit unnecessary and repetitive workups.

 The majority of the most frequent users were insured through Medicaid or Medicare; about one-third were uninsured. This belies the stereotype that frequent users are uninsured and may indicate a high incidence of disabling conditions, especially among the nonelderly population. It also implies that the Medicaid expansion in the Patient Protection and Affordable Care Act (PPACA), which targets low-income individuals, may not by itself decrease frequent ED use.

 The conditions most prominent among this group of frequent ED users are acute symptoms such as pain, nausea/vomiting, altered consciousness, and respiratory complaints. Their visits are also related to chronic diseases such as HIV infection, hypertension, drug and alcohol abuse/withdrawal, and diabetes. This combination of acute and chronic complaints indicates the importance of arranging appropriate followup to cover complex medical and behavioral health concerns as well as social challenges.

Homelessness was the most consistently unifying characteristic among these 20 patients. Interestingly, when ICD-9 codes were used to screen for homelessness, lack of housing was found to be one of the top five diagnoses in six patients. When these same patients' unique identifiers were compared with the homeless service records, 18 of the 20 patients were identified as having experienced homelessness during the year. This might suggest that ED staff members' familiarity with certain frequent visitors may cause them to not document housing status or recognize the role that lack of housing may play in their medical conditions.

Homeless patients with complex medical issues face significant challenges in managing their health and securing consistent community-based care that helps stabilize their chronic and acute conditions and thus lessen the need for subsequent ED visits. Very-low-income individuals who are homeless tend to receive services at centers offering comprehensive care encompassing medical and behavioral health care as well as access to social services, but these services often fall short of providing stable housing.

## 5. Limitations

This study was limited to a small subset of total ED users. Care must be taken in generalizing to the larger group of frequent ED users and to frequent ED users in other areas. Data were not collected on admission rates or ED wait times, thereby limiting conclusions on ED and inpatient resource use among this population. Triage scores were used to suggest visit acuity but can only approximate actual acuity of illness. Triage methodology is different in each department; we attempted to ameliorate this by combining triage scores into broader categories. Coding may have been incomplete or inaccurate, limiting conclusions drawn about each patient's overall health. Care providers' familiarity with these individuals may have resulted in abbreviated documentation. Information on providers' awareness of these patients' use patterns and the impact of this awareness on clinical and resource decisions was not examined.

 Our definition of homelessness was intentionally broad, including anyone who presented to one of two homeless service systems during the year of the study. It is not known if the two patients not identified as homeless simply did not use the mainstream homeless services that informed our study. Patients were not necessarily homeless at the time they presented to the ED.

 Data from three EDs formed the basis of this study. These three departments lie within a few miles of each other, and they are not the only departments in the immediate area or the larger metropolitan region. Visits to other EDs would be unaccounted for in this study.

## 6. Conclusion

The frequent ED users identified in this study have significant medical problems. Their homelessness appears to be a unifying characteristic and may be a contributing factor to the frequency of their ED visits. Further evaluation of this patient group might demonstrate whether interventions such as intensive case management, access to housing programs that include health services, and targeted efforts to manage complex coexisting chronic diseases would improve the overall health of this population and decrease ED utilization.

## Figures and Tables

**Table 1:** Characteristics of top 20 ED users.

Patient no.	Total no. of visits	No. of EDs	Age	Homeless?	Triage levels*			No. of ICD-9 codes	Top five diagnoses^†^	Primary insurance
					High (1, 2)	Moderate (3)	Low (4, 5)			
1	185	2	55	Yes	29	112	9	341	Lack of housing (59), pain in limb (32), alteration of consciousness (26), abdominal pain (23), nausea/vomiting (14) 45.1% of all codes	Medicaid

2	182	3	49	Yes	25	107	37	97	HIV (18), pruritic disorder (9), cough/chest pain (9), skin rash (6), dizziness (4), dermatitis (4), asymptomatic HIV infection (4) 55.6% of all codes	Medicare

3	168	3	41	Yes	44	101	15	506	Nondependent abuse of drugs (105), alteration of consciousness (98), alcohol withdrawal (92), hypertension (60), and unspecified alcohol dependence (51) 80.2% of all codes	Medicaid

4	148	3	43	Yes	41	73	7	246	Lack of housing (28), back pain (25), HIV infection (21), pain/swelling in limb (21), nondependent abuse of drugs (15) 44.7% of all codes	Medicaid

5	132	3	56	Yes	25	88	13	367	Pain/swelling in limb (33), shortness of breath/cough (31), lack of housing (30), back pain (28), HIV infection (21) 38.9% of all codes	Medicare

6	105	2	62	No	66	30	8	412	Shortness of breath/chest pain (117), diabetes (59), hypertension (49), coronary atherosclerosis (40), alteration of consciousness (33) 72.3% of all codes	Medicare

7	100	2	55	Yes	22	35	24	209	Alteration of consciousness (42), alcohol dependence (39), lack of housing (30), nondependent abuse of drugs (22), noncompliance with medical treatment presenting hazards to health (15) 70.8% of all codes	Uninsured

8	99	2	43	Yes	4	65	11	158	Respiratory symptoms (28), headache (20), abdominal pain (15), nausea/vomiting (12), nondependent abuse of drugs (10) 53.8% of all codes	Uninsured

9	97	3	56	Yes	7	75	7	290	Drug withdrawal (32), HIV infection (30), opioid dependence (23), nausea/vomiting (23), diabetes (22) 44.8% of all codes	Medicaid

10	92	3	56	Yes	16	51	20	294	Diabetes (60), respiratory symptoms (33), pain in limb (23), nausea/vomiting (15), asthma (12) 48.6% of all codes	Uninsured

11	87	2	56	Yes	17	45	13	239	Lack of housing (40), myalgia/pain in limb (23), alcohol abuse (16), alcohol dependence (12), joint pain (11) 42.6% of all codes	Uninsured

12	87	3	53	Yes	9	60	11	240	Asthma (33), respiratory abnormalities (21), pain in limb (21), depression (13), disorders of the joint (11) 41.2% of all codes	Uninsured

13	82	3	43	Yes	22	41	13	310	Nausea/vomiting (40), abdominal pain (38), HIV (31), alteration of consciousness (23), respiratory symptoms (23) 50% of all codes	Medicaid

14	82	3	42	Yes	8	66	7	263	Hypertension (30), headache (23), respiratory symptoms (22), alterations of consciousness (19), myalgia/pain in limb (19) 42.9% of all codes	Medicaid

15	80	3	36	Yes	5	66	5	259	Nausea/vomiting (41), abdominal pain (41), hypertension (28), bipolar disorder (25), chronic ulcer of skin (20) 59.8% of all codes	Medicaid + Uninsured

16	76	3	30	No	55	15	3	167	Sickle cell disease (115), pain in limb (31), depression (10) 93.4% of all codes	Medicaid

17	70	1	59	Yes	15	48	5	236	Hypertension (42), bronchitis (29), asthma (26), pain in limb (24), shortness of breath (24) 61.4% of all codes	Uninsured

18	70	3	40	Yes	16	26	10	105	Nondependent alcohol abuse (20), depressive disorder (14), schizophrenia (11), other unspecified alcohol dependence (9), unspecified nonpsychotic mental disorder (8), joint pain in lower leg (8) 66.6% of all codes	Medicaid

19	70	3	68	Yes	19	20	30	324	Chest pain (64), diabetes (41), back pain (23), syncope (22), hypertension (16) 51.2% of all codes	Medicare

20	67	2	23	Yes	16	29	14	152	Lack of housing (31), acute upper respiratory infection (19), headache (17), acute nasopharyngitis (common cold) (16), acute pharyngitis (11) 61.8% of all codes	Uninsured

Total	2,079	1 : 1 2 : 6 3 : 13	Ave: 48.3 Mean: 51	18 of 20	461 (24.6%)	1,153 (61.4%)	262 (14%)	5,215		Medicaid: 8.5 Uninsured:: 7.5 Medicare: 4

*Triage totals do not match visit totals because of missing data (9.8% of table sample).

^†^Includes the number of instances this ICD-9 code was cited during the calendar year.

**Table 2:** Most prevalent diagnoses among 20 frequent ED users.

Diagnosis indicated by ICD-9 Code	No. of patients
Limb pain	9
Lack of housing	6
Alteration of consciousness	6
HIV infection	5
Nausea/vomiting	5
Hypertension	5
Drug abuse	5
Alcohol abuse/withdrawal	4
Diabetes	4
Abdominal pain	4
Respiratory symptoms	4
